# Integrative transcriptomic analysis for linking acute stress responses to squamous cell carcinoma development

**DOI:** 10.1038/s41598-020-74051-7

**Published:** 2020-10-14

**Authors:** Tran N. Nguyen, Kimal Rajapakshe, Courtney Nicholas, Leticia Tordesillas, Erik A. Ehli, Christel M. Davis, Cristian Coarfa, Elsa R. Flores, Sally E. Dickinson, Clara Curiel-Lewandrowski, Kenneth Y. Tsai

**Affiliations:** 1 Department of Tumor Biology, H. Lee Moffitt Cancer Center and Research Institute, Tampa, FL 33612 USA; 2 Department of Computational Biomedicine, Vingroup Big Data Institute, Hanoi, Vietnam; 3 Department of Molecular Biology, Baylor College of Medicine, Houston, TX 77030 USA; 4 Department of Immunology, University of Texas MD Anderson Cancer Center, Houston, TX 77030 USA; 5 Avera Institute for Human Genetics, Sioux Falls, SD 57108 USA; 6 Department of Molecular Oncology, H. Lee Moffitt Cancer Center and Research Institute, Tampa, FL 33612 USA; 7 Donald A. Adam Melanoma and Skin Cancer Center of Excellence, H. Lee Moffitt Cancer Center and Research Institute, Tampa, FL 33612 USA; 8 Department of Pharmacology, University of Arizona Cancer Center, Tucson, AZ USA; 9 Department of Dermatology, University of Arizona Cancer Center, Tucson, AZ USA; 10 Department of Anatomic Pathology, H. Lee Moffitt Cancer Center and Research Institute, 12902 Magnolia Dr, SRB-4, Tampa, FL 33612 USA

**Keywords:** Cancer genomics, Squamous cell carcinoma

## Abstract

Cutaneous squamous cell carcinoma (cuSCC) is the second most common skin cancer and commonly arises in chronically UV-exposed skin or chronic wounds. Since UV exposure and chronic wounds are the two most prominent environmental factors that lead to cuSCC initiation, we undertook this study to test whether more acute molecular responses to UV and wounding overlapped with molecular signatures of cuSCC. We reasoned that transcriptional signatures in common between acutely UV-exposed skin, wounded skin, and cuSCC tumors, might enable us to identify important pathways contributing to cuSCC. We performed transcriptomic analysis on acutely UV-exposed human skin and integrated those findings with datasets from wounded skin and our transcriptomic data on cuSCC using functional pair analysis, GSEA, and pathway analysis. Integrated analyses revealed significant overlap between these three datasets, thus highlighting deep molecular similarities these biological processes, and we identified Oncostatin M (OSM) as a potential common upstream driver. Expression of OSM and its downstream targets correlated with poorer overall survival in head and neck SCC patients. In vitro, OSM promoted invasiveness of keratinocytes and cuSCC cells and suppressed apoptosis of irradiated keratinocytes. Together, these results support the concept of using an integrated, biologically-informed approach to identify potential promoters of tumorigenesis.

## Introduction

Skin cancers, of which 20% are cutaneous squamous cell carcinomas (cuSCC), comprise the most common group of malignancies in humans. Because its incidence is rapidly increasing, cuSCC poses a significant public health and economic burden^1^. UV radiation is recognized as the main environmental etiological agent that drives initiation and progression of sporadic cuSCC^2,3^. Although not as common, a subset of high-risk cuSCC displaying aggressive biological behavior occurs in chronic wounds sometimes associated with burns (so-called Marjolin’s ulcers) or genodermatoses such as recessive dystrophic epidermolysis bullosa (RDEB). In fact, metastatic cuSCC is the main cause of death among RDEB patients^4,5^. Since UV exposure and chronic wounds are the two most prominent environmental factors that lead to cuSCC initiation, we sought to ask whether signatures of more acute molecular responses of skin to UV exposure and wounding would potentially overlap with signatures of established cuSCC. This notion is predicated on the previously established concept that molecular signatures of acute UV exposure are relevant to subsequent pathway alterations in established tumors^6,7^. For example, the canonical *TP53*-driven transcriptional program and c-Jun-N-terminal kinase (JNK) stress responses are activated in response to UV exposure and these pathways are established as tumor suppressive^8–10^.

**Figure 1:**
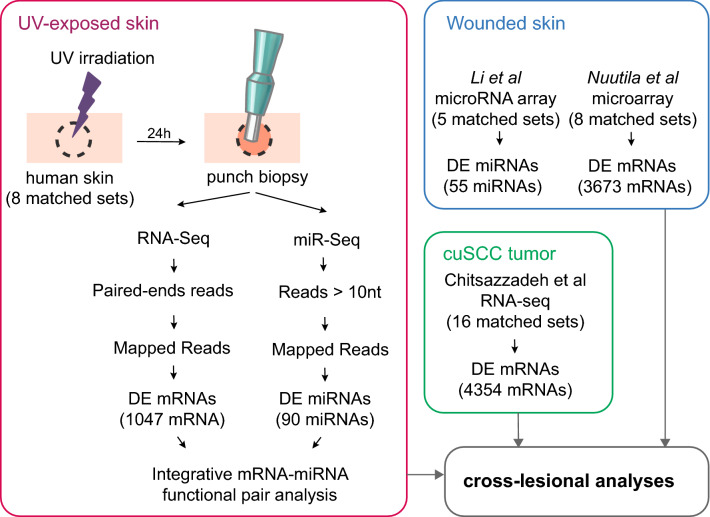
Data-driven target identification from gene expression profiles across disease development. Integration of gene expression profiles for acutely UV-exposed and unexposed skin, wounded and normal skin, cuSCC tumors and normal skin. Samples from tumors and normal skin were separately analyzed for gene and miRNA expression using microarrays, RNA-seq and miRNA-seq. miRNAs and mRNAs expression were then paired by functional pair analyses and characterized by GSEA. (Adobe Illustrator CS6, https://www.adobe.com/).

In the current study, we conducted miR-omic and transcriptomic profiling using next generation sequencing (Illumina) on 8 sets of acute UV-exposed normal sun-protected human skin, by integrating differentially expressed (DE) miRNAs and mRNAs in functional pair analysis (Fig. 1). We further predicted enriched biological pathways related to acute UV exposure using GSEA^11^. We then integrated miRNA and RNA profiles of acute UV-exposed skin with previously reported transcriptional profiles of wounded skin^12,13^ (Fig. 1). Our combinatorial analyses demonstrated that acute UV-exposed skin (at 24 h) and wounded skin (at 3 day) shared remarkable similarity in their miRNA and RNA profiles. Finally, we compared the miRnome and transcriptomes of acute UV-exposed skin and wounds to those of cuSCC. We found that not only did DE genes from these distinct biological contexts overlap significantly but they also shared similarly enriched pathways that converged on Oncostatin M (OSM) and TNFα.

Here, we demonstrated a direct effect of OSM on keratinocyte and cuSCC migration and invasion, and we additionally defined a role for OSM on UV-induced apoptosis. Overall, through integrated transcriptomic analyses of related data sets, we identified a consensus set of deregulated molecular pathways and a potential driver of cuSCC development.

## Methods

### Cell culture

Normal human epidermal keratinocytes (NHEK) were purchased from Lonza and maintained in KGM-Gold Keratinocyte Basal Medium (Lonza). HaCaT cells, an immortalized human keratinocyte line cell was obtained from Dr. Norbert Fusenig (German Cancer Research Center) and maintained in DMEM/F12 (1:1), supplemented with 10% heat-inactivated fetal bovine serum, and 100 U/mL penicillin and 100 μg/mL streptomycin. Cells were maintained at 37 °C in a 95% air and 5% CO_2_ humidified incubator. Medium was replaced every 2–3 days and cells were routinely subjected to mycoplasma tests. Only mycoplasma free cells were used in experiments. All lines were STR profiled to confirm distinct identities.

### MED determination and irradiation of human subjects

These methods were as previously reported^6,7^. The solar simulated light was generated using a Multiport UV Solar Simulator Model 600 (Solar Light Co., Philadelphia, PA, USA) with a spectral irradiance consisting of 8.7% UVB and 91.3% UVA. The dose of emission was precisely regulated to be limited to UVA and UVB spectra (290–390 nm). In brief, the minimal erythemal dose (MED) of UV (290–320 nm) was determined for each subject using this device as the smallest dose that produced confluent erythema on the buttocks with four distinct borders 22–24 h post-UV exposure. Following exposure, the test sites were covered until evaluations were completed. After determination of the MED for each individual, the contralateral buttock was exposed to four times that MED. A 4 mm skin punch biopsy was collected from one buttock at baseline prior to UV exposure and additional 4 mm punch biopsies were removed at 30 min, 1 h and 24 h post-irradiation. These samples were collected under IRB Protocol entitled “Analysis of UVA and UVB Induced Molecular Target Expression and Activation in Human Skin: Cross-Validation with Known Murine Targets, Project No. 04-0478-01 (Previously HSC# 04-107) at UACC and sequenced and studied under IRB Protocol LAB08-0750 at MD Anderson Cancer Center. The University of Arizona and University of Texas MD Anderson Cancer Center Institutional Review Boards both approved the study and written informed consent was obtained from all study participants. We additionally confirm that that all research was performed in accordance with relevant guidelines/regulations. Healthy subjects recruited for this study are 18 years or older and have Fitzpatrick skin types either II (burns easily, tans poorly) or III (burns moderately, tans gradually). All subjects were Caucasian and non-Hispanic. The ages ranged from 39.6 to 84.4 years in age (average 60.2). Of the eight patients whose samples were used, the range was 39.6–84.4 years in age (average 58.5).

### Human samples (cuSCC, actinic keratoses, skin)

All human tissues were studied under a MD Anderson Cancer Center Institutional Review Board-approved protocol (LAB08-0750). All human tissues were obtained from patients who provided written informed consent and who had no history of immunosuppression. We additionally confirm that that all research was performed in accordance with relevant guidelines/regulations. These samples were validated by histological analysis and processed using standard methods to yield both high-quality DNA and RNA (RIN > 8.0). Some of these samples have been previously analyzed and reported^14^; in this cohort, an additional 24 samples from 8 patients (matched sets of normal skin, actinic keratoses (AK), and cuSCC) are included.

### UV-induced apoptosis assay

The solar simulated light was generated using a UV Solar Simulator UV SOL (Oriel, Newport Technologies, Irvine, CA, USA). The spectrum of light generated by the solar simulator consisted of 8.0% UVB and 92.0% UVA. The light was metered by measuring broadband UVB. The dose of emission was precisely regulated to be limited to UVA and UVB spectra (280–400 nm). Cells were pre-treated with 80 ng/mL OSM and vehicle for 1 h, irradiated using a solar simulator at the dose of 6 J/cm^2^ (metered using broadband UVB). At 24 h post-irradiation, both floating and adherent cells were collected and stained with FITC Annexin V and PI (BioLegend). A negative control of unstained cells and positive controls of cells treated with 3% formaldehyde for 30 min on ice and stained with either PI or Annexin V were used to gate the flow cytometer for viable, early apoptotic, late apoptotic, and necrotic cell populations. Samples were subjected to FACS analyses (FACS Canto II, BD Bioscience). Data were obtained and analyzed using and FlowJo software (v.10.5.2, BD Bioscience).

### Scratch motility assay

Cells were seeded in 96-well plates and pre-treated with and without 80 ng/mL OSM for 1 h and during the wound healing process. The wound maker (Essen Bioscience) was used to make uniform horizontal scratches in the cell monolayer of each well and the cells treated with 10 µM mitomycin C (M4287; Sigma Aldrich) to inhibit proliferation. Cells were continuously imaged for 48 h following wound formation using IncuCyte live cell analysis system (Essen Bioscience). IncuCyte ZOOM software (v.2016B) was used to calculate the wound healing time.

### Transwell invasion and migration assay

Transwell chambers (Millipore) were used for the cell migration assay according to the manufacturer's protocol. DMEM medium supplemented with 10% fetal bovine serum (FBS) was the chemoattractant. For the migration assay, cells were incubated for 24 h, and those adherent to the upper membrane were removed. Cells that migrated or invaded through the membrane were fixed and stained with 0.1% crystal violet. For the invasion assay, the procedure was identical, except that upper chamber membranes were pre-coated with 100 µL Matrigel (BD Biosciences, Franklin Lakes, NJ, USA). The cells were photographed and counted across five fields, and each experiment performed in triplicate.

### RNA-Seq (NGS) analysis

We used next generation sequencing (RNAseq) for samples of UV-exposed skin and cuSCC tumors. The mRNA-seq paired-end reads were aligned to the human reference genome, GRCh37/hg19, using the TopHat2 alignment software^15,16^. The overlaps between aligned reads and annotated genomic features, such as genes/exons were counted using HTSeq software^17^. Hierarchical clustering analysis, using the Pearson correlation coefficient as the distance metrics and the complete linkage, and principal component analysis (PCA) were performed using the R statistical system. Genes significantly different between the control and different time points of acute UV treatment were determined using the R package DESeq^18^ (p ≤ 0.05). Since multiple genes were tested simultaneously, the Benjamini–Hochberg method was used to control false discovery rate (FDR). For further integration of mRNAs and miRNAs, and detection of enriched transcription factor targets, we used a cutoff of q ≤ 0 0.05 and fold change exceeding 1.25×.

### smallRNA-Seq (NGS) analysis

We used next generation sequencing (smallRNA-seq) for samples of UV-exposed skin and cuSCC tumors. This work was performed with collaboration with laboratory of Dr. Preethi Gunaratne, PhD (University of Houston, Biology & Biochemistry). As previously described^14^, Illumina small RNA adapter sequences were trimmed from the reads, and reads of length below 10nt or ending in homopolymers of length 9 nt or above were discarded. Total usable number of reads for each sample was calculated. The reads were mapped to the miRBase v21^19^ reference using blastn v 2.2.26 where we requested exact matches to the miRNA reference, and allowed matches also to at most 4 base pairs flanking the mature miRNA sequence; the abundance of each expressed miRNA was quantified as a fraction of the usable reads, and expressed as parts per million We determined differentially expressed miRNAs imposing a fold-change of 1.25 × and t-test comparison (p ≤ 0.05) using the R statistical system. We employed principal component analysis (PCA) to examine sample structure; further visualization of miRNA expression in one or multiple comparisons was carried out using the R statistical system.

### Integrative mRNA-miRNA functional pair analysis

We determined enriched miRNA–mRNA pairs using the SigTerms methodology. Essentially, by applying a one-sided Fisher exact test and using the TargetScan^20^ predicted miRNA targets, we determined the miRNAs for which the gene targets are significantly enriched (FDR-adjusted q ≤ 0.25; fold-change ≥ 1.25×) in the gene signature, separately for the human specimens and the mouse samples^14^. Finally, we determined the conserved enriched miRNAs alongside the SCC progression model, and the conserved miRNA–mRNA pairs conserved alongside the SCC progression model. Conserved enriched miRNA–mRNA pairs were visualized using the Cytoscape software (v.3.5.0).

### Gene expression omnibus data mining

We retrieved two transcriptome profiles from GEO which is a public genomics database, allowing users to investigate gene expression profiles of interest. The GSE28914 is a microarray dataset of human wound in a GPL570 Affymetric Human Genome U133 Plus v2 array platform, which contains 25 biopsies from different time points (right after, 3 days and 7 days) of the wound healing process. The GSE2822 is a microarray dataset of cultured NHEK with OSM in a GPL8300 Affymetrix Human Genome U95 v2 Array Platform, which contains NHEK samples underwent OSM treatment at 4 time points (1 h, 4 h, 24 h and 48 h).

Processed gene expression dataset was downloaded and *limma* 3.42 package^21^ was used to determine the differentially expressed genes between normal and investigated tissues. p ≤ 0.05 and fold-change ≥ 1.25 × were considered as the cutoff values. Batch effects were accounted for using COMBAT implemented in *inSilicoMerging* 1.0.8 package^22^.

### Kaplan–Meier analysis

The overall survival curves were investigated using the Kaplan–Meier method with the log-rank test. We set the high and low gene expression level groups by the median value. The overall survival plot was graphed with the hazard ratio (HR) based on the Cox PH Model and the p value. The log-rank p value was calculated with < 0.05 considered statistically significant. The analysis was implemented using the web-based tool PROGgene^23^. In brief, the gene names were entered into the database, the HNSC dataset GSE65858 with *TP53* mutation (to avoid HPV+ cases) was selected, after which survival plots were generated.

### Statistical analyses

The presented data were all obtained from at least three independent experiments. The error bars in all figures are standard errors of the mean (SEM). The visualization of graphed data was performed using GraphPad Prism (v.8.0) (GraphPad Software) and a parametric unpaired *t*-test primarily used. Significance is denoted in the figures as the following: * (p < 0.05), ** (p < 0.01) and *** (p < 0.001).

## Results

### A small number of miRNAs drive changes in gene expression in acute UV-exposed skin

While prior studies have assessed changes in miRNA expression in keratinocytes following short-term UV exposure^24^, linked miRNA–mRNA expression has yet to be characterized in UV-irradiated normal human skin. To investigate this, we exposed normally sun-protected human buttock skin to a single acute dose of four times the mean erythemal dose (MED) of solar simulated light and obtained specimens in RNAlater pre- and post-UV in 8 healthy subjects. RNA-Seq and miRNA-seq (NGS, Illumina) was performed to measure the changes in mRNA and miRNA expression at 1 h and 24 h post-UV exposure (Supplementary Table 1). We found that 1 h after UV exposure, there were 1 upregulated and 4 downregulated miRNAs. At the same time point, there were 552 upregulated genes (72% of expressed genes) and 215 downregulated genes (28% of expressed genes). On the other hand, in 24 h post-UV skin samples, there were 50 upregulated miRNAs and 39 downregulated miRNAs compared to pre-exposure skin. In addition, we found that there were 1363 upregulated genes (45% of expressed genes) and 1659 downregulated genes (55% of expressed genes).

**Figure 2:**
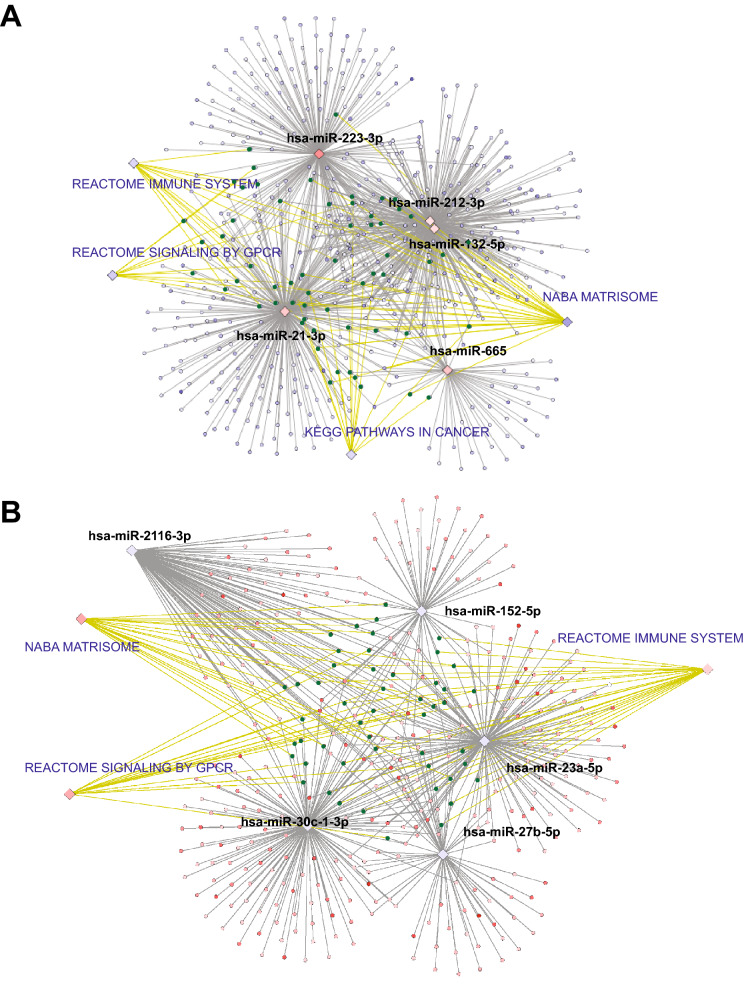
Deregulated pathways and miRNAs associated with acute UV response in unexposed human skin. Computationally integrated networks of miRNAs (black text), genes (red and blue dots) and pathways (blue text). Genes that are part of the gene sets were marked green dots. The grey lines connect miRNAs to their predicted target genes. The yellow lines connect genes to the gene sets. The network of anticorrelated miRNA genes was computed with the information of target prediction derived from DIANA-microT, TargetScan, and miRNA.org. Two hundred and forty-five interactions were found among 16 miRNAs and 84 genes. The Cytoscape tool was applied to display the interconnections between features. (**A**) Associations of miRNAs and target genes based on functional pair analyses (fold change higher than 5 is shown). (**B**) Associations of genes and pathways based on GSEA (enrichment score over 5 is shown). (Cytoscape 3.7.1^47^. https://cytoscape.org/, Adobe Illustrator CS6, https://www.adobe.com/).

Notably, the miR-mRNA functional pair analysis showed that 10 (or 13%) of the most differentially-expressed (DE) miRNAs were predicted to target 906 (or 30%) of the most DE genes. Gene Set Enrichment Analysis (GSEA) of this miRNA-targeted mRNA subset showed that upregulated and downregulated miRNAs indirectly targeted common pathways (through their predicted target genes) (Fig. 2). These analyses suggested that shortly after UV exposure, there was change in a small number of miRNAs that led to large-scale changes in mRNAs, potentially affecting immune responses and the skin microenvironment.

### Similarity of transcriptomic signatures of acute UV-exposed skin, acute wounds and cuSCC tumors

Since cuSCC often develops in chronically UV-exposed skin and non-healing wounds are a significant risk factor, we asked the question of whether acute responses to these stressors could inform our understanding of the early molecular events contributing to cuSCC formation. We therefore focused our subsequent analyses on comparing the transcriptomes of acutely UV-exposed skin, subacutely (3 day) wounded skin, and cuSCC tumors^14^. Prior reports have showed that molecular signatures of acute UV exposure drive subsequent pathway alterations in established tumors due to chronic UV exposure^6,7^. As an example, *TP53* and stress kinase responses are well known to be activated in response to UV exposure; yet these responses are downregulated in tumors by mutation or epigenetic means, or they are regarded as tumor suppressive pathways^8,9,25–28^.

**Figure 3:**
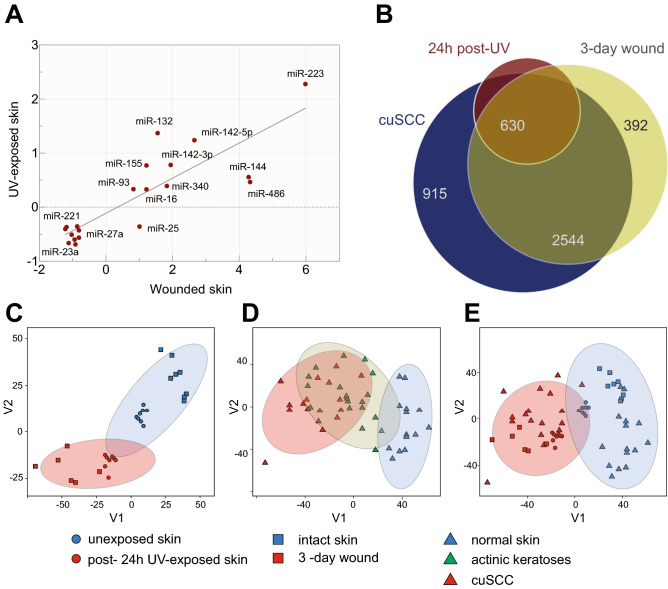
Integrated analyses show overlapping in gene expression profiles across disease development. (**A**) Strong correlation (R^2^ = 0.7) in miRNA levels in post UV-exposed and wounded skin. Both samples were collected 24 h after UV-exposure and wounding. The dots represent miRNAs with log_2_ fold-change higher or lower than 0.5 (p < 0.05). (**B**) Overlapping gene expression profiles of cuSCC tumors, post-UV exposed and wounded skin. The area of displayed circles is proportional to the numbers of genes. (**C**) Correlation in gene expression profiles of 24 h post-UV exposed (red circle) vs. unexposed skin (blue circle)^48^. (**D**) Correlation in gene expression profiles of normal skin (blue triangle), actinic keratosis (green triangle) and cuSCC tumors (red triangle). Gene expression profiles of actinic keratoses (AK) shows significant overlap with both normal skin and cuSCC tumors, as previously reported^14^. (**E**) Correlation of 24 h post-UV exposed skin, 3-day post-wounded skin (red square), cuSCC tumors, and their normal counterparts (blue circle, triangle and square). [R (3.5.0)^48^, https://www.r-project.org/, Adobe Illustrator CS6, https://www.adobe.com/].

We used published data based upon the Taqman miRNA array profiling of miRNA expression changes during the inflammatory phase of human skin wound healing in 5 healthy donors 0 and 24 h post-injury^12^. Interestingly, we found that the expression pattern of miRNA in acute UV-exposed skin was strongly correlated with acute wound healing skin at 24-h time point (Fig. 3A). The pro-inflammatory miRNA, miR-223, was the most upregulated miRNA in both data sets after 24 h, suggesting a common inflammatory response at this time point following UV-exposure and injury. Among the upregulated miRNAs, miR-132 has been characterized in wound-healing but not in the acute UV response^12,29^.

To study whether the mRNA expression profiles of UV-exposed skin, wounded skin and cuSCC tumors also overlapped, we again used published transcriptomic data set on wounded skin^13^. This study focused on 8 burn patients who underwent skin grafting and from whom 25 skin biopsies were obtained immediately before and after harvesting and during the wound healing process 3 and 7 days thereafter. The samples were subjected to genome-wide microarray analysis (Affymetrix Human Genome U133 Plus 2.0 GeneChip Array and Illumina Human HT‐12 GeneChip). We observed that 3-day healing wounds had 3081 DE genes (p-value < 0.05, log_2_FC < − 0.5 or log_2_FC > 0.5) while 7-day wounds had 2682 DE genes (p-value < 0.05, log_2_FC < − 0.5 or log_2_FC > 0.5). GSEA showed that 89% of enriched gene sets in 7-day wound overlapped with those in 3-day wound, while only 63% of enriched gene sets in 3-day wound overlapped with those in 7-day wound. We concluded that the 3-day wound profile might capture a wider spectrum of potentially relevant biological pathways. Since the 3-day sample is a closer time point to the 24-h UV-exposed skin time point, we decided to use 3-day wound data for integrative analyses with the 24-h post-UV skin.

Initially, we assessed the overall shared DE genes between 24 h post-UV skin, 3-day wound and cuSCC tumors. We found that 84% of DE genes in UV-exposed skin overlapped with DE genes in cuSCC, and 87% of DE genes in 3-day wounds overlapped with DE genes in cuSCC (Fig. 3B). To better visualize the overall changes across the three data sets, we performed principal components analysis (PCA)^30^. The transcriptome data projected the two distinct clusters of the UV-exposed skin, wounded skin and cuSCC tumors and their normal control counterparts (Fig. 3C–E). First, we projected the transcriptomes of the acute UV and subacutely (3 day) wounded skin datasets (Fig. 3C), demonstrating overlap between normal unirradiated and intact skin and significant overlap between acutely irradiated and subacutely (3 day) wounded skin. We also observed that the gene expression profiles of UV-exposed skin overlapped with those of actinic keratosis (AK, a precancerous transitional stage between normal skin and cuSCC) while the gene expression profiles of wounds were most closely related to those of cuSCC (Fig. 3C–D). Similarly, we projected the transcriptomes of normal skin, AKs and cuSCC tumors and observed that the transcriptomes of AKs spanned those of normal skin and cuSCC (Fig. 3D). This observation is aligned with previous studies from our lab where we showed the transcriptomic profiles of AKs spanned the spectrum of normal skin to cuSCC^14^. Finally, when these signatures were combined into one plot (Fig. 3E), one could readily group the normal skin, intact skin, and unirradiated skin in one area (blue) and the cuSCC, wounded skin, and irradiated skin in a distinct one (red). Overall, these results showed that there was a strong correlation between the mRNA and miRNA transcriptomic profiles of UV-exposed skin, subacutely wounded (3 day) skin and cuSCC tumors, thus reinforcing the notion that these biological contexts have important molecular commonalities.

### Identification of enriched pathways

**Figure 4:**
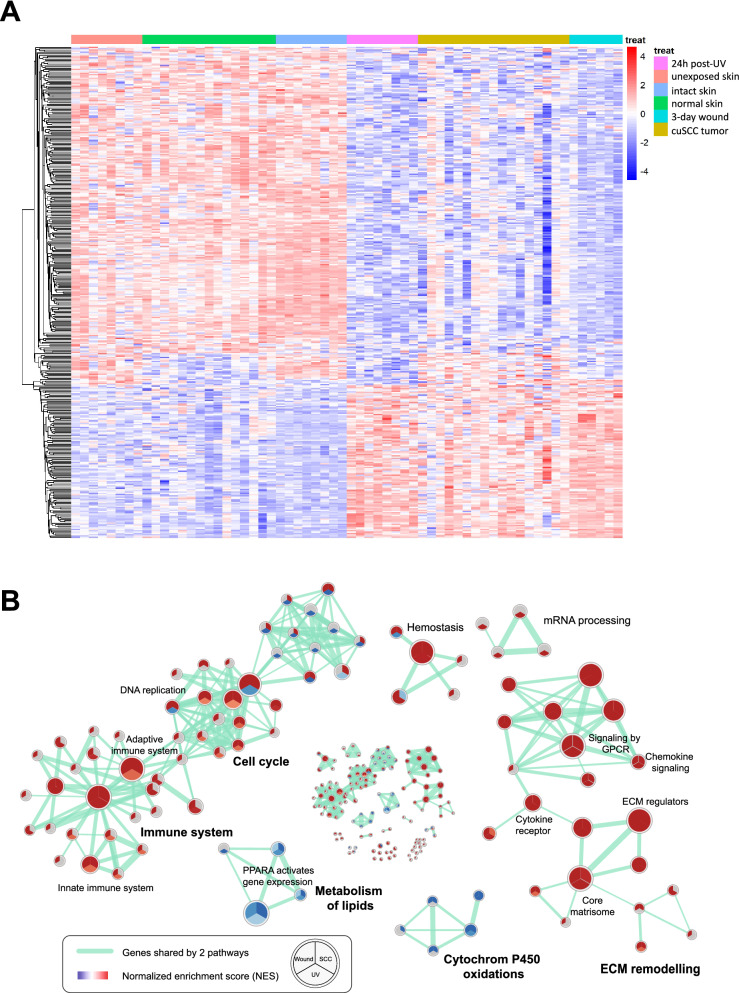
(**A**) Heatmap highlighting differentially-expressed genes across the three key comparisons: acutely UV-exposed skin vs. unexposed, cuSCC tumors vs. normal skin, and subacutely (days) wounded skin vs. intact skin. These results highlight a large set of common DE among all three comparisons. (**B**) Canonical pathway network changes in UV-exposed skin, wounded skin and cuSCC tumors predicted by GSEA. Then associated pathway map was generated using Enrichment Map Plugin^49^ developed for Cytoscape^50^. Significant terms with a false discovery rate less than 0.01 are shown. Each node (or circle) represents a gene set. Node size is proportional to prevalence of the Gene Ontology term in each gene expression profiles and edge width is proportional to the degree of gene overlap between two nodes. Some node names within a group were replaced with a general term for clarity. Each circle is divided into three sections where each section represents one of three datasets. Blue color represents downregulated pathways. Red color represents upregulated pathways (p = 0.05). (Cytoscape 3.7.1^47^. https://cytoscape.org/, Adobe Illustrator CS6, https://www.adobe.com/).

**Figure 5:**
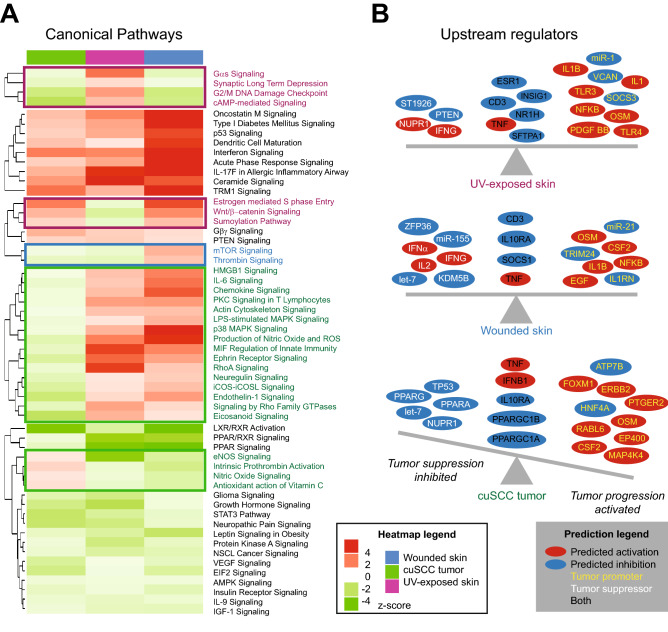
IPA analysis reveal important common changes in UV-induced skin, wounded skin and cuSCC tumors. (**A**) Unique and common Ingenuity Pathway Analysis (IPA) canonical pathways shared by the three data sets^48^. (**B**) Identified upstream regulators in three data sets by IPA analysis. [R (3.5.0)^48^, https://www.r-project.org/, Adobe Illustrator CS6, https://www.adobe.com/].

The global functional impact of acute UV-exposure vs. subacute (3 day) wounded vs. cuSCC^14^ was determined by using 3 approaches: integrated GSEA analyses (Fig. 4A,B), upstream regulator predictions through IPA (Fig. 5A), and canonical pathways prediction also through IPA (Fig. 5B). Using pathway analyses with multiple approaches allowed us to better identify central key pathways that were affected.

We first looked at overlapping DE across three datasets and were able to identify significant overlap between all three comparisons, highlighting our ability to quickly capture molecular commonalities in these three contexts (Fig. 4A). Next, we sought to identify unique and common pathways affected by acutely UV-exposed skin, subacutely (3 day) wounded skin and cuSCC tumors using GSEA. We focused on the common pathways because these are the pathways that were modified following UV-exposure and wound-healing that persisted in cuSCC tumors. We reasoned that by focusing on these pathways, we could identify ways to predict early development of cuSCC. Here, we observed that ECM-related pathways, GPCR signaling, cytokine and chemokine signaling, immune system signaling and cell cycle signaling were enriched (Fig. 4B), suggesting that these pathways were activated in common in acutely UV-exposed skin, subacutely (3 day) wounded skin, and cuSCC tumors.

Using Ingenuity Pathway Analysis (IPA), we identified both unique and common pathways and initially focused on the common pathways modified in acutely UV-exposed skin, subacutely (3 day) wounded skin and cuSCC tumors because of the above rationales. We found that five signaling pathways were activated, including immune response pathways such as Oncostatin M, interferon, and acute phase response signaling (Fig. 5A,B). The IPA upstream regulator analysis examines how many known targets of transcription regulators are present in the provided data sets with the direction of changes taken into consideration. Here, the group of upstream regulators identified suggested that in cuSCC and in irradiated skin, there were more activated tumor promoters than inhibited tumor suppressors, which suggested a pro-tumorigenic environment (Fig. 5A), consistent with the notion that UV-exposed and wounded skin are more at-risk for developing skin cancer. On the other hand, in subacutely (3 day) wounded skin, the number of activated tumor promoters and inhibited tumor suppressors (and vice versa) appears more balanced. Nevertheless, two soluble factors that were commonly predicted in all three data sets were Oncostatin M (OSM) and TNF-α (Fig. 5B). Even though TNF-α inhibitors might increase risk of cuSCC due to immunosuppression^31,32^, the role of TNF-α in cuSCC development remains inconclusive.

**Figure 6:**
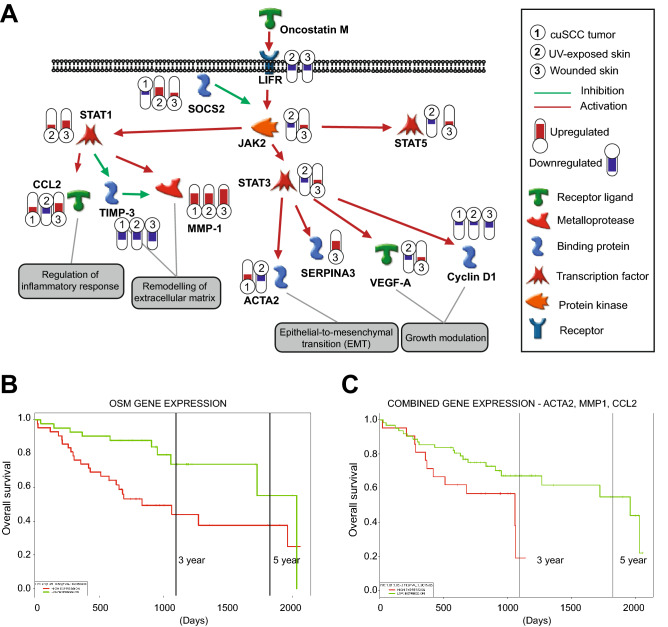
Oncostatin M is a potential regulator of cuSCC development. (**A**) Oncostatin M downstream networks. GeneGO pathway generator identifies possible downstream molecular targets and cellular processes regulated by Oncostatin M. (**B**) Kaplan–Meier survival analysis of OSM gene expression in HNSCC patients with *TP53* mutation. In this cohort, high expression of OSM is associated with shorter overall survival (High: median survival = 831 days; Low: median survival = 2033). Kaplan–Meier curves were generated following bifurcation of gene expression at median (high: n = 42, low: n = 41). P-values were calculated according to the log-rank test. (**C**) Kaplan–Meier survival analysis of ACTA2, MMP1, CCL2 gene expression in HNSCC patients with *TP53* mutation. In this cohort, high expression of OSM is associated with shorter overall survival (high: median survival = 1057 days; low: median survival = 1962). Kaplan–Meier curves were generated following bifurcation of gene expression at median (high: n = 42, low: n = 41). P-values were calculated according to the log-rank test. (MetaCore GeneGo, https://portal.genego.com/ and Adobe Illustrator CS6, https://www.adobe.com/).

We further investigated reported evidence concerning the effects of OSM on human skin by searching the GEO database for ‘Oncostatin’ and ‘skin’ related datasets. We found that the dataset GSE2822^33^ included the transcriptomic profile of OSM-treated NHEK. Using GSEA and Enrichment maps, we integrated this transcriptome with the other transcriptomes of UV-exposed skin, wound and cuSCC tumors based on the shared canonical pathways. We found that pathways related to cell cycle and extracellular matrix regulation were enriched which is similar to what we have observed previously (Supplementary Fig. S1, Figs. 4B, 5). This suggested that OSM treatment of NHEK could induce similar pathways observed in UV-exposed skin, wounds and cuSCC tumors (Fig. 6). Finally, we noted that OSM expression was upregulated during acute UV exposure and in subacute (3 day-old) wounds by 2.1 and 7.8-fold, respectively (Supplementary Table 2). Therefore, we decided to focus on characterizing potential roles for OSM in promoting phenotypes associated with tumor development.

### Oncogenic activities of Oncostatin M

Oncostatin M (OSM) is a gp130 family cytokine^34^ elaborated by a variety of immune cells in multiple biological contexts including hematopoiesis, bone remodeling, and inflammatory processes^35,36^. OSM can activate multiple signal transduction kinase pathways^37^ and can inhibit keratinocyte differentiation^38^. However, there is not yet data reporting OSM involvement in cuSCC development^35^. To address this, we gathered in silico evidence on how OSM could have a role in tumorigenesis. Pathway mapping using MetaCore software (v.6.30, Clarivate Analytics) suggested that OSM can exert its effect through TIMPs and MMPs to regulate ECM remodeling, through Cyclin D1 and VEGF to modulate growth, and through CCL2 to regulate inflammatory responses (Fig. 6A). Next, we explored a previously published transcriptome data set where human keratinocytes were treated with OSM at multiple time points^33^. Here, GSEA showed that gene sets involved in DNA repair, adhesion, cell cycle and transcription were enriched (Supplementary Fig. S1).

Next, we sought to validate whether OSM and its downstream targets were clinically relevant in squamous cell carcinoma. Due to the paucity of survival data linked to molecular profiles in cuSCC, we used survival data from head and neck squamous cell carcinoma (HNSCC) patients who carry *TP53* mutation because genetically, this type of carcinoma most closely related to cuSCC^14^. The Kaplan–Meier survival curves showed that high OSM expression related with shorter overall survival time in comparison with low OSM expression (p-value = 0.005) (Fig. 6B). In addition, survival analysis based on expression of the OSM downstream signaling components *ACTA2*, *MMP1*, *CCL2* as identified by GeneGO analysis, showed that high expression of these genes related to shorter overall survival time (Fig. 6C) in patients with *TP53-* mutant HNSCC. These results suggest that OSM signaling may be clinically relevant in SCC and that OSM can modulate biological processes that are important for tumorigenesis.

### Oncostatin M increased motility and suppressed UV-induced apoptosis in keratinocytes

**Figure 7:**
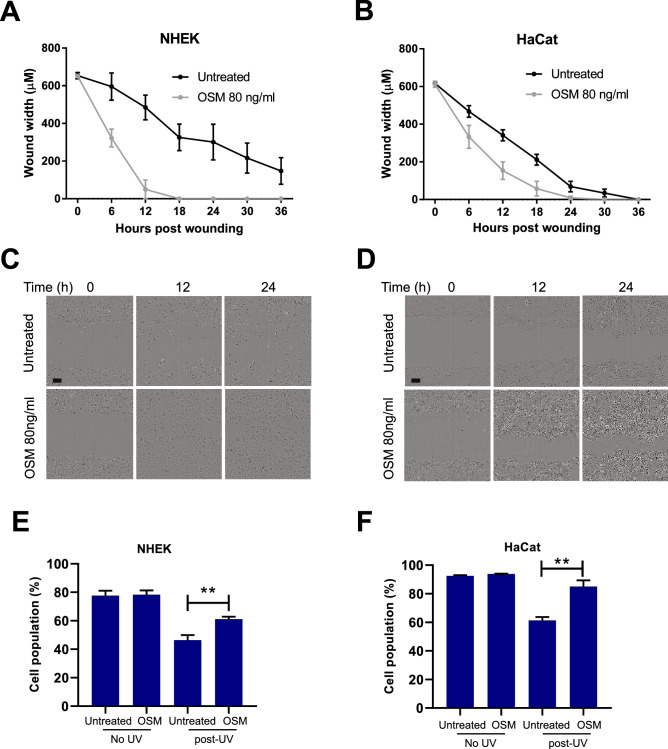
Oncostatin M increases cellular motility and post-UV viability of NHEK and HaCat cells. (**A**–**D**) Cell motility shown by wound healing/scratch assay. Shown are the representative light microscope images showing healing of wounds in monolayers of NHEK and HaCat treated with or without OSM 80 ng/mL at 0, 12 and 24 h after the scratch was created. The rate of migration calculated by the width of the wound in NHEK and HaCat by Incucyte ZOOM software (V2018B). Triplicate experiments were conducted, and representative results are shown. Data were analyzed using GraphPad Prism 8.0 and a parametric unpaired *t*-test was performed where *p < 0.05, **p < 0.01, ***p < 0.001. (**E**,**F**) Detection of apoptosis of NHEK cells following UV irradiation. Cells were starved in low serum (1% FBS) media overnight, treated with OSM 80 ng/mL for 1 h. Then cells were UV-irradiated and maintained with or without OSM 80 ng/mL for 24 h before being subjected to the combined Annexin V binding-PI staining assay. Plots in (**E**) represent the mean ± SEM percentage of viable (PI and annexin negative), apoptotic (Annexin-positive) and necrotic (PI-positive and Annexin-negative). Triplicate experiments were conducted, and representative results are shown. Data were analyzed using GraphPad Prism 8.0 and a parametric unpaired *t*-test was performed where *p < 0.05, **p < 0.01, ***p < 0.001.

Based on the pathways mapped by MetaCore and the impact on outcomes in HNSCC (Fig. 6), we aimed to study the effect of OSM on keratinocyte migration and UV-induced apoptosis. For this, we treated NHEK and HaCat cells with recombinant OSM. We found that at OSM increased the migratory capacity of NHEK and HaCat relative to untreated cells in an in vitro wound-healing assay (Fig. 7A–D). At 12 h post-wounding, quantification of the cells in the wounded area showed a 9.8 ± 2.6-fold decrease in wound width in NHEK (p = 0.0007) and 2.2 ± 0.3-fold decrease in HaCaT cells as compared to control-treated cells (p = 0.001). To study the effect of OSM on the invasion capability of cuSCC cells, we performed transwell cell invasion assays on IC1. Here, we observed that OSM-treated IC1 cells increased their invasiveness to 8.74 ± 1-fold compared to untreated IC1 control (p = 0.017) (Supplementary Fig. S2). We were unable to perform transwell cell invasion assay on NHEK and HaCat because of the relatively non-invasive nature of the cells.

Having established the association between OSM and cell mobility, we further explored the possibility of a direct effect of OSM on UV-induced apoptosis in keratinocytes. To address this, we performed apoptosis assay on UV-irradiated and OSM-treated NHEK. 24 h post-UV-irradiation, the percentage of healthy OSM-treated keratinocytes (Annexin V^−^, PI^−^) significantly increased compared to untreated cells (Fig. 7E,F, Supplementary Fig. S2). In detail, percentage of healthy NHEK increased by 1.31 ± 0.11-fold (p = 0.004) and in HaCaT, percentage of healthy cells increased by 1.39 ± 0.09-fold (p = 0.009). Altogether, these findings suggested that OSM can promote pro-tumorigenic phenotypes by enhancing cell mobility and resistance to apoptosis.

## Discussion

Here, we used multiple gene expression and miRNA expression profiles to show that the transcriptomic deregulation observed in cuSCC is closely related to the early changes in acutely UV-exposed skin and subacutely (3 day) wounded skin, which reflect environmental exposures related to two prominent high-risk settings for cuSCC development (Figs. 1, 3). These signatures also show likely contributions from inflammatory mediators perhaps elaborated by immune infiltrates, which are collectively well-understood to be critically important contributors to cancer development in humans and in animal models. We report the first comprehensive transcriptional in-vivo profile of the response to UV exposure in human skin. Importantly, we showed molecular evidence for the transcriptomic similarity between UV-exposed skin and subacutely (3 day) wounded skin (Figs. 3, 4). Currently, UV and wounding have been treated as two independent events in non-melanoma skin cancer development. Our findings provide unique evidence showing that transcriptomes of UV-exposed skin, wounded skin and cuSCC overlap and we propose that key drivers of tumorigenesis can be identified through such analyses.

UV-exposure and skin wounding triggers similar transcriptional responses in human skin through several common pathways (Figs. 3, 4). First, there is activation in pathways associated with inflammatory responses. Although inflammation is the second stage of wound healing, it is unexpected that we observed similar response in acute UV-exposed skin. These inflammatory responses might have resulted from the release of a variety of regulatory mediators including cytokines and chemokines following UV-exposure or skin injuries^39,40^. Here, at the UV irradiated sites or wounded sites, components of innate immune system such as macrophages are also recruited. It has been previously shown that macrophage electrotaxis is mostly dependent on Rho family small GTPases^41^. Interestingly, from GSEA, we observed enrichment of GPCR signaling that might have been precursors to macrophage recruitment.

OSM has been reported to promote a variety of pathologies, including skin inflammation, and various types of squamous cell carcinoma^35,36,42–44^. Integrated analyses using multiple data sets and pathway analysis platforms suggested that Oncostatin M signaling was activated early in UV-exposed skin and wounded skin as well as in cuSCC tumors (Figs. 4, 5). These initial data prompted us to characterize OSM involvement in keratinocytes. Here, OSM-enhanced motility is in accordance with previous in vitro studies^42,45,46^. Such tumor-promoting effects were regulated through JAK/STAT3 pathways^44,46^. In the context of cuSCC, we reasoned that OSM can act through both STAT1 and STAT2 to induce cell motility through upregulation of ACTA2 and matrix metalloproteinases together with down-regulation of tissue inhibitors of metalloproteinases (Fig. 6). We showed that OSM suppressed UV-induced apoptosis in human keratinocytes (Fig. 7), suggesting a further tumor promoting activity in cuSCC development. Here, even though the molecular targets through which the OSM pathway ultimately acts will need further validation, our integrated pathway analyses suggest that VEGF and cyclins are potentially important markers (Fig. 6).

We propose that this type of integrated analysis incorporating key biological information and clinical contexts might be effectively leveraged to identify key pathways in cancer development and identify potential vulnerabilities. In our case, we integrated genomic data from the two biological contexts of acute UV exposure and subacutely (3 day) wounded skin to identify key common changes that overlap with signatures of established cuSCC. By combining information from seemingly disparate datasets, we are able to arrive at biological processes potentially amenable to intervention in cancer. Further comprehensive molecular validation of the multiple pathways identified in our analysis will be required to demonstrate this conclusively.

## Supplementary information

Supplementary Table 1.

Supplementary Table 2.

Supplementary Figures.

## Data Availability

The datasets used and/or analyzed during the current study are available from the corresponding author on reasonable request.
