# Supplementary material for: Integrative transcriptomic analysis for linking acute stress responses to squamous cell carcinoma development
Source: Sci Rep. 2020 Oct 14;10:17209. doi: 10.1038/s41598-020-74051-7 (PMC7560606; doi:10.1038/s41598-020-74051-7)
Supplement: Supplementary file 3 — Supplementary Figures. [file 41598_2020_74051_MOESM3_ESM.pdf]

SUPPLEMENTARY FIGURES:

Integrative Transcriptomic Analysis For Linking Acute Stress Responses to Squamous Cell Carcinoma Development

Tran N. Nguyen[1,2], Kimal Rajapakshe[3], Courtney Nicholas[4], Leticia Tordesillas[1], Erik Ehli[5], Christel

Davis[5], Cristian Coarfa[3], Elsa R. Flores[6,7], Sally Dickinson[8], Clara Curiel-Lewandrowski[9], Kenneth

Y. Tsai[1,7,10]

Departments of Tumor Biology[1], Molecular Oncology[6], and Anatomic Pathology[10]
Donald A. Adam Melanoma and Skin Cancer Center of Excellence[7]
H. Lee Moffitt Cancer Center and Research Institute, Tampa, FL 33612

Department of Computational Biomedicine[2]
Vingroup Big Data Institute, Hanoi, Vietnam

Department of Molecular Biology[3]
Baylor College of Medicine, Houston, TX 77030

Department of Immunology[4]
University of Texas MD Anderson Cancer Center, Houston, TX 77030

Avera Institute for Human Genetics[5]
Sioux Falls, SD 57108
Departments of Pharmacology[8] and Dermatology[9]
University of Arizona Cancer Center, Tucson, AZ

Correspondence:

Kenneth Y Tsai, MD, PhD
Departments of Anatomic Pathology & Tumor Biology
H. Lee Moffitt Cancer Center & Research Institute
12902 Magnolia Dr
SRB-4
Tampa, FL 33612

Email: Kenneth.tsai@moffitt.org
Phone: (813) 745-4864

Figure S1

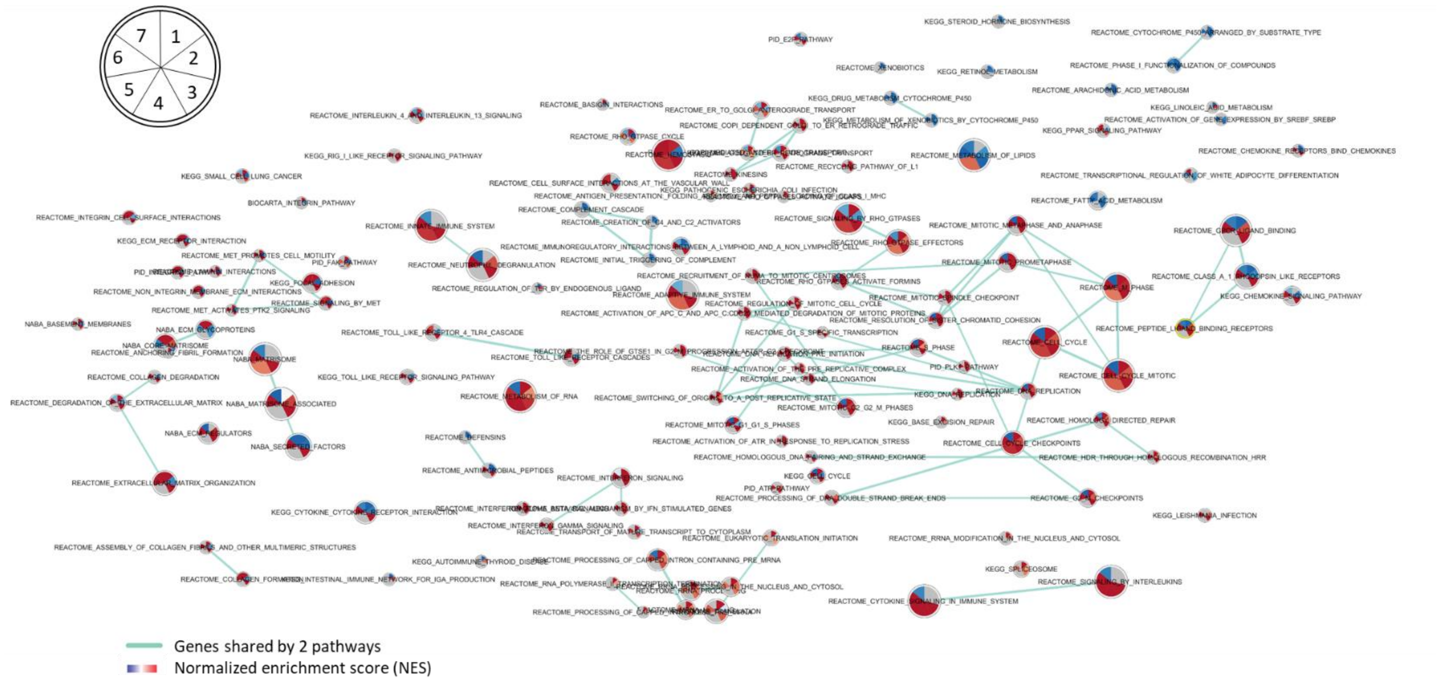

Association in the transcriptomes of OSM-treated NHEK, UV-exposed skin, wounded skin and cuSCC tumors.

GSEA canonical pathway network in OSM-treated NHEK, UV-exposed skin, wounded skin and cuSCC tumors. Associated pathway map was generated using Enrichment Map Plugin (Merico, Isserlin et al. 2010) developed for Cytoscape (Smoot, Ono et al. 2011). Significant terms with a false discovery rate less than 0.01 are shown. Each node (circle) represents a gene set. Node size is proportional to prevalence of the Gene Ontology term in each gene expression profiles and edge width is proportional to the degree of gene overlap between two nodes. Each circle is divided into seven sections where each section represented a dataset. The dataset in the circle is in the following order: (1) 1h-post-OSM-treated NHEK, (2) 24h-post-OSM-treated NHEK, (3) cuSCC tumors, (4) 1h-post-UV-exposed skin, (5) 1h-post-UV-exposed skin, (6) acutely-wounded skin and (7) 3-day wound. Blue color represents downregulated pathways. Red color represents upregulated pathways ($p = 0.02$). (Cytoscape 3.7.1. https://cytoscape.org/, Adobe Illustrator CS6, https://www.adobe.com/)

Figure S2

A

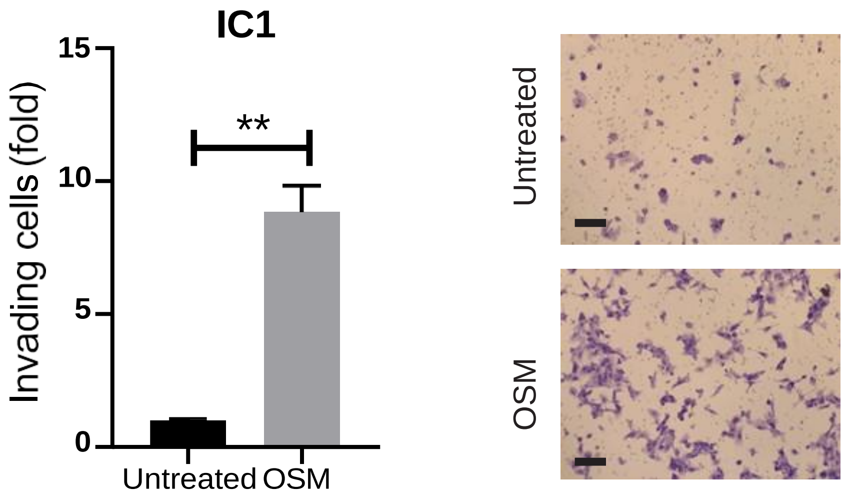

B

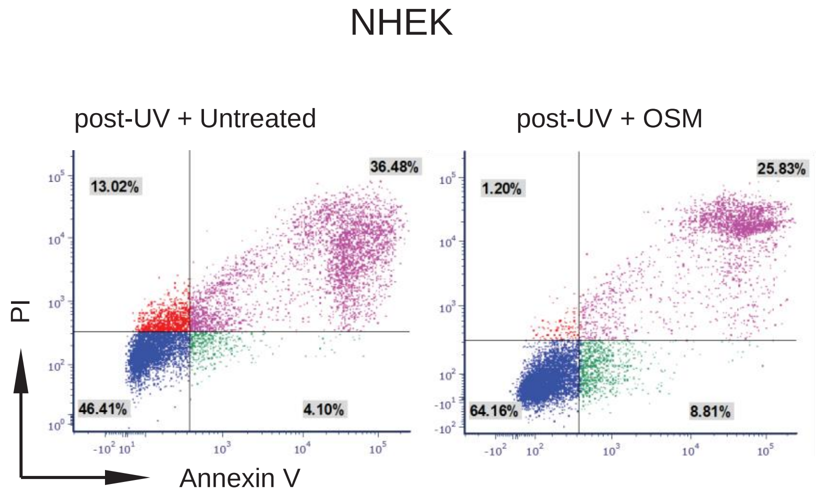

C

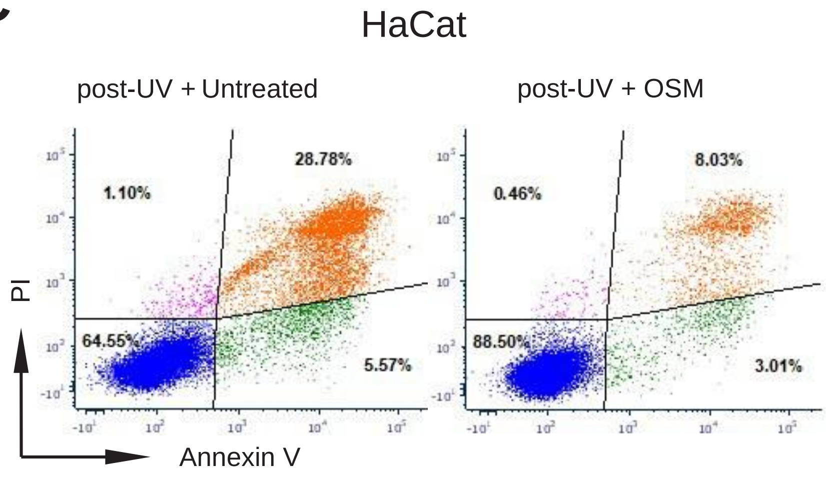

Effect of OSM on augmenting tumorigenic phenotypes of keratinocytes

(A) Transwell cell invasion assay show increases in IC1 invasion after OSM treatment. IC1 were treated with or without OSM 80 ng/ml for 24 hours before collected for tranwell assay. Cells were cultured for 24 hr before the membranes were collected, stained and counted. **(B-C)** Detection of apoptosis of NHEK and HaCat cells following UV irradiation. Cells were starved in low serum (1% FBS) media overnight, treated with OSM 80 ng/ml for 1 hour. Then cells were UV-irradiated and maintained with or without OSM 80 ng/ml for 24 hours before being subjected to the combined Annexin V binding-PI staining assay. Triplicate experiments were conducted, and representative results are shown. Data were analyzed using GraphPad Prism 8.0 and a parametric unpaired *t*-test was performed where *$p < 0.05$, ** $p < 0.01$, *** $p < 0.001$.
